# Supplementary material for: Marine Polyether Phycotoxin Palytoxin Induces Apoptotic Cell Death via Mcl-1 and Bcl-2 Downregulation
Source: Mar Drugs. 2023 Apr 6;21(4):233. doi: 10.3390/md21040233 (PMC10143546; doi:10.3390/md21040233)

Figure S1: Calculated IC50 values of different human cancer cell lines incubated with palytoxin at various concentrations between 2 and 8 hours. Data are the mean of SD ± of three independent experiments.

Leukemia cell lines:

K562	IC_{50} (pM)
2h	52.1 ± 5.9
4h	32.8 ± 7.8
6h	28.5 ± 7.3
8h	24.9 ± 5.0

Jurkat	IC_{50} (pM)
2h	12.8 ± 6.0
4h	13.5 ± 2.1
6h	8.8 ± 1.0
8h	8.7 ± 1.0

U937	IC_{50} (pM)
2h	24.8 ± 1.7
4h	27.3 ± 8.1
6h	14.0 ± 6.1
8h	9.0 ± 0.7

Solid tumor cell lines:

A549	IC_{50} (pM)
2h	93 ± 9.8
4h	46.5 ± 5.8
6h	30.8 ± 3.7
8h	31.5 ± 2.7

PC3	IC_{50} (pM)
2h	93.2 ± 20.4
4h	72.1 ± 9.9
6h	31.5 ± 2.8
8h	35.9 ± 4.6

MCF7	IC_{50} (pM)
2h	136.4 ± 30.9
4h	98.9 ± 9.2
6h	88.6 ± 7.8
8h	72.1 ± 8.8

SH-SY5Y	IC_{50} (pM)
2h	62.4 ± 7.3
4h	48.6 ± 5.9
6h	32.1 ± 3.6
8h	23.1 ± 3.9

Figure S2: Palytoxin down-regulates the expression of anti-apoptotic Bcl-2 family proteins in solid tumor cell lines. Western blot analysis of anti-apoptotic Bcl-2 family protein expression levels in A549 (lung cancer), PC3 (prostate cancer), MCF7 (breast cancer), and SH-SY5Y (neuroblastoma) cells.

A549

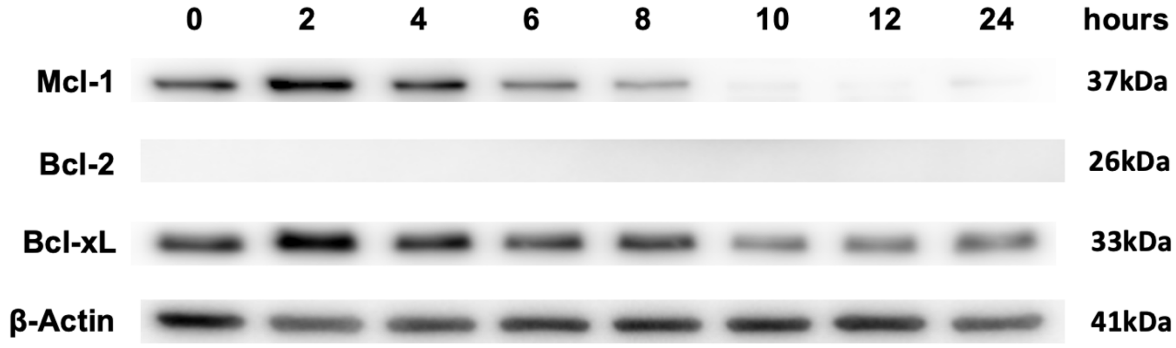

PC3

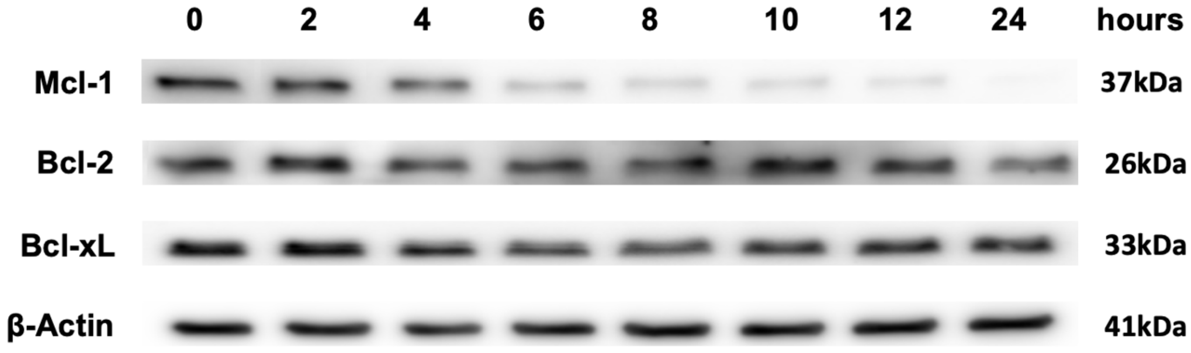

MCF7

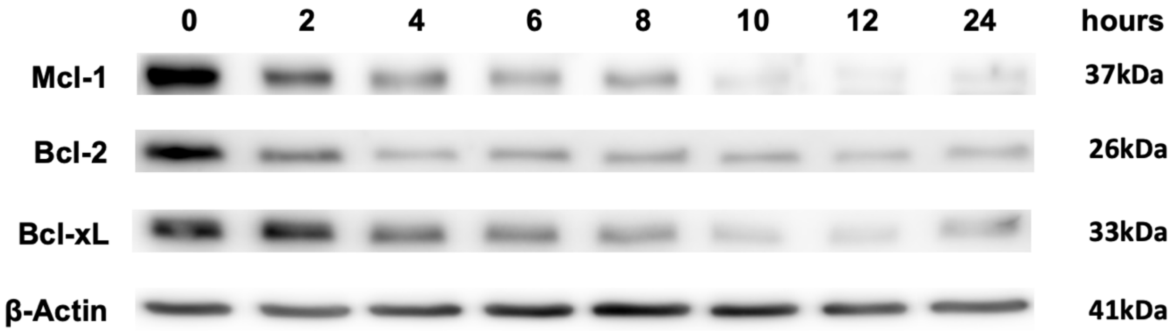

SH-SY5Y

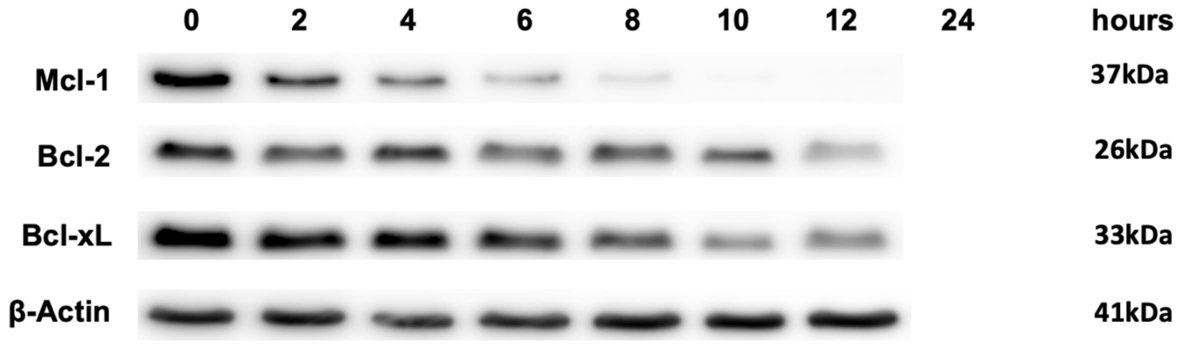
0
2
4
6
8
10
12
24
hours
Mcl-1
Bcl-2
Bcl-xL
β-Actin
37kDa
26kDa
33kDa
41kDa

Figure S3: Palytoxin reduces the colony-forming ability in PC3 prostate cancer cells. Palytoxin showed an inhibitory effect on colony formation in a dose-dependent manner. All data represent the mean ± SD. of three independent experiments. Statistical significance was assessed as **p<0.01 versus control.

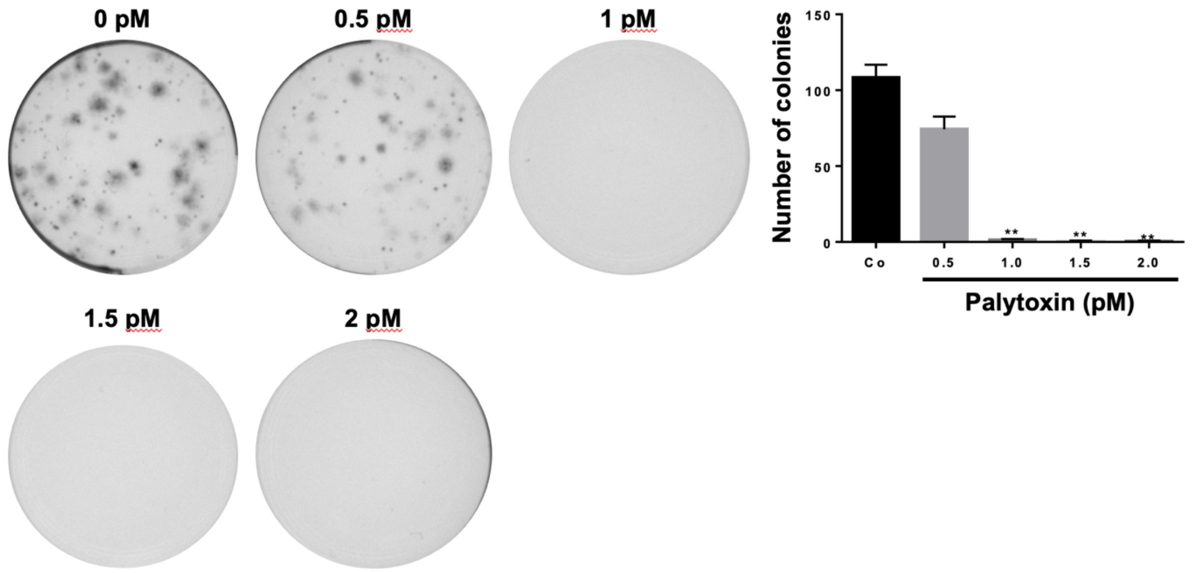

Figure S4: Palytoxin inhibited PC3 prostate cancer spheroid formation in a dose-dependent manner. Statistical significance was assessed as *p<0.05 and ***p<0.0001 versus control.

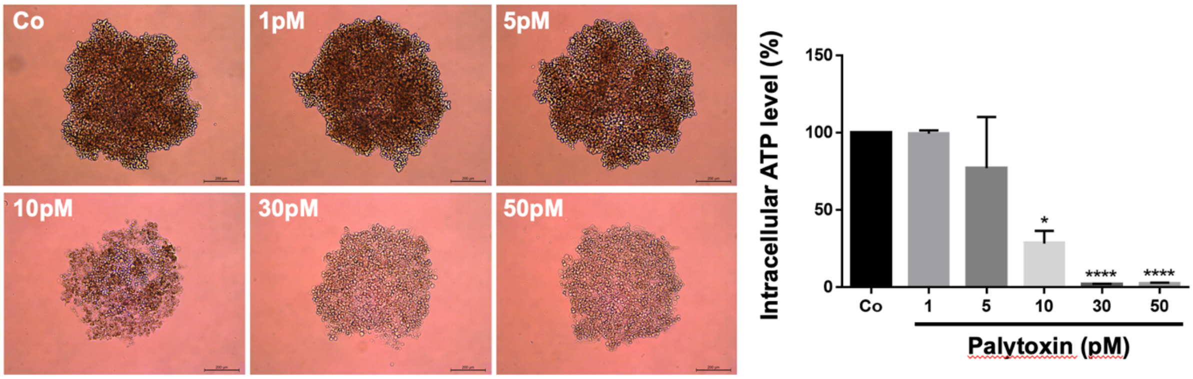

Figure S5: Palytoxin inhibits tumor-forming ability in *in vivo* zebrafish xenografts. Fluorescently-labeled leukemia cells were treated with palytoxin at 5, 10, and 30 pM for 6 hrs and then injected into the zebrafish yolk sac. A total of nine fish were obtained per condition. Top: bright field, middle: CM-Dil, bottom: merge. PBS injection was used as a control for injection toxicity.

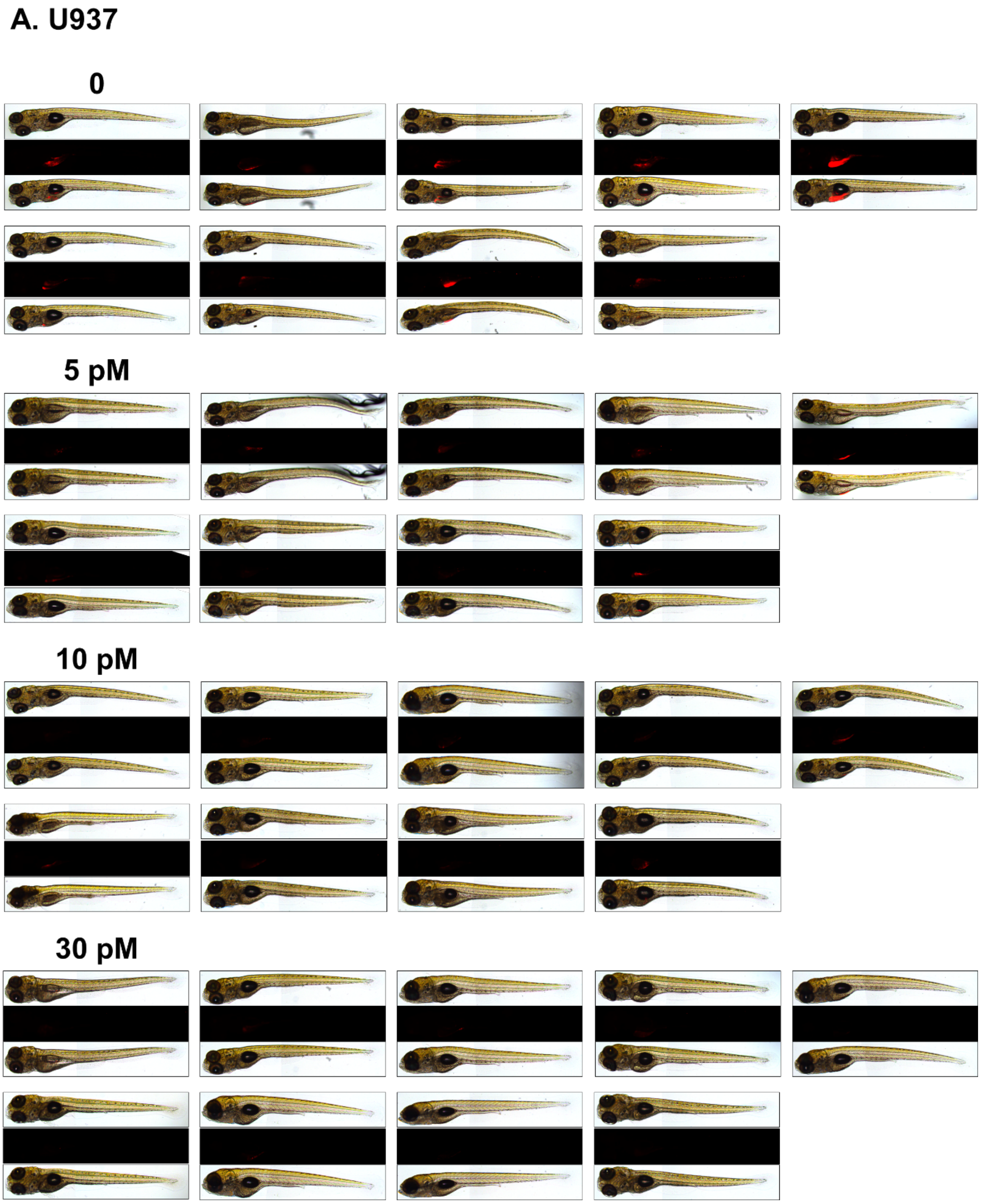

B. HL60

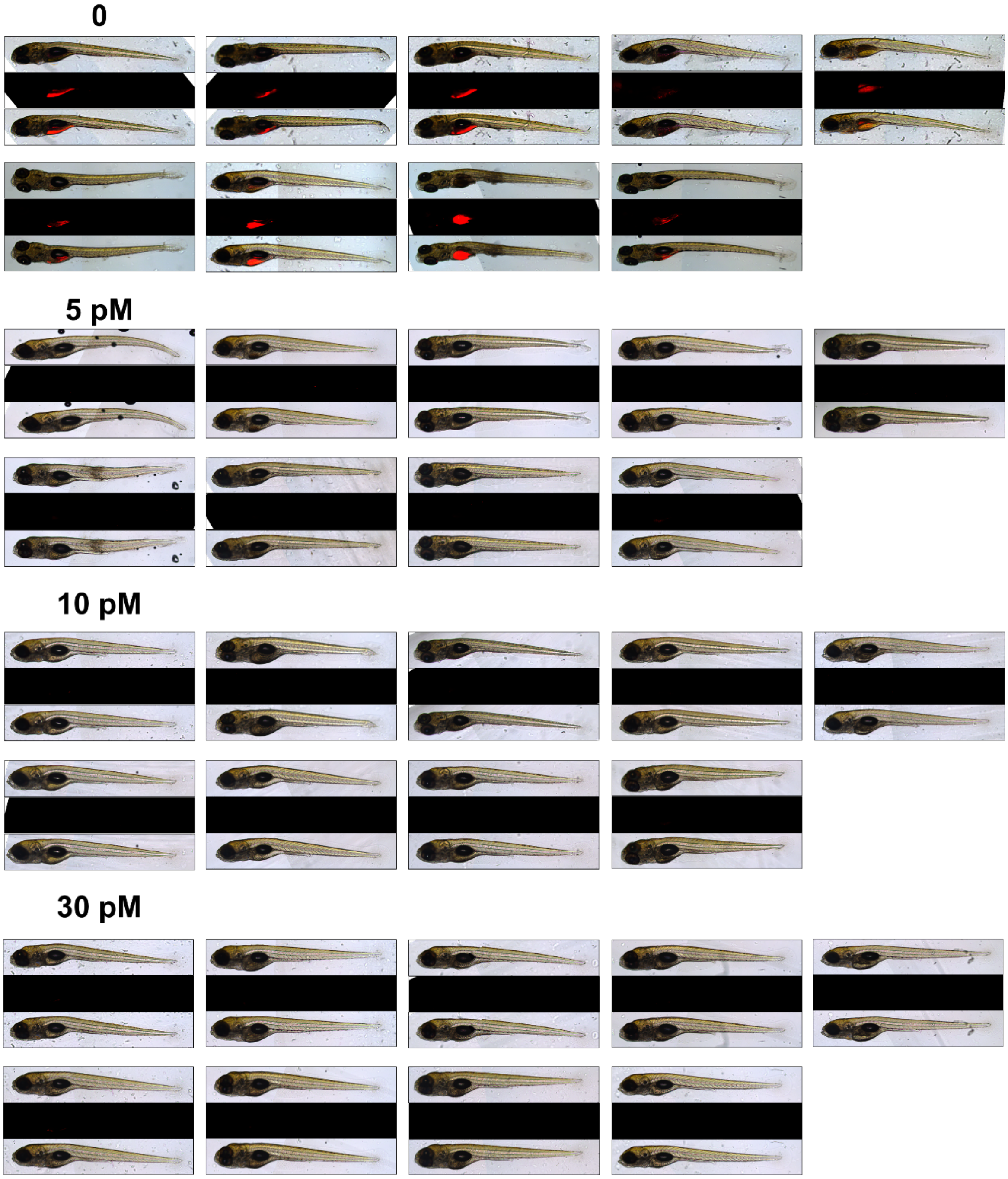

C. KG-1

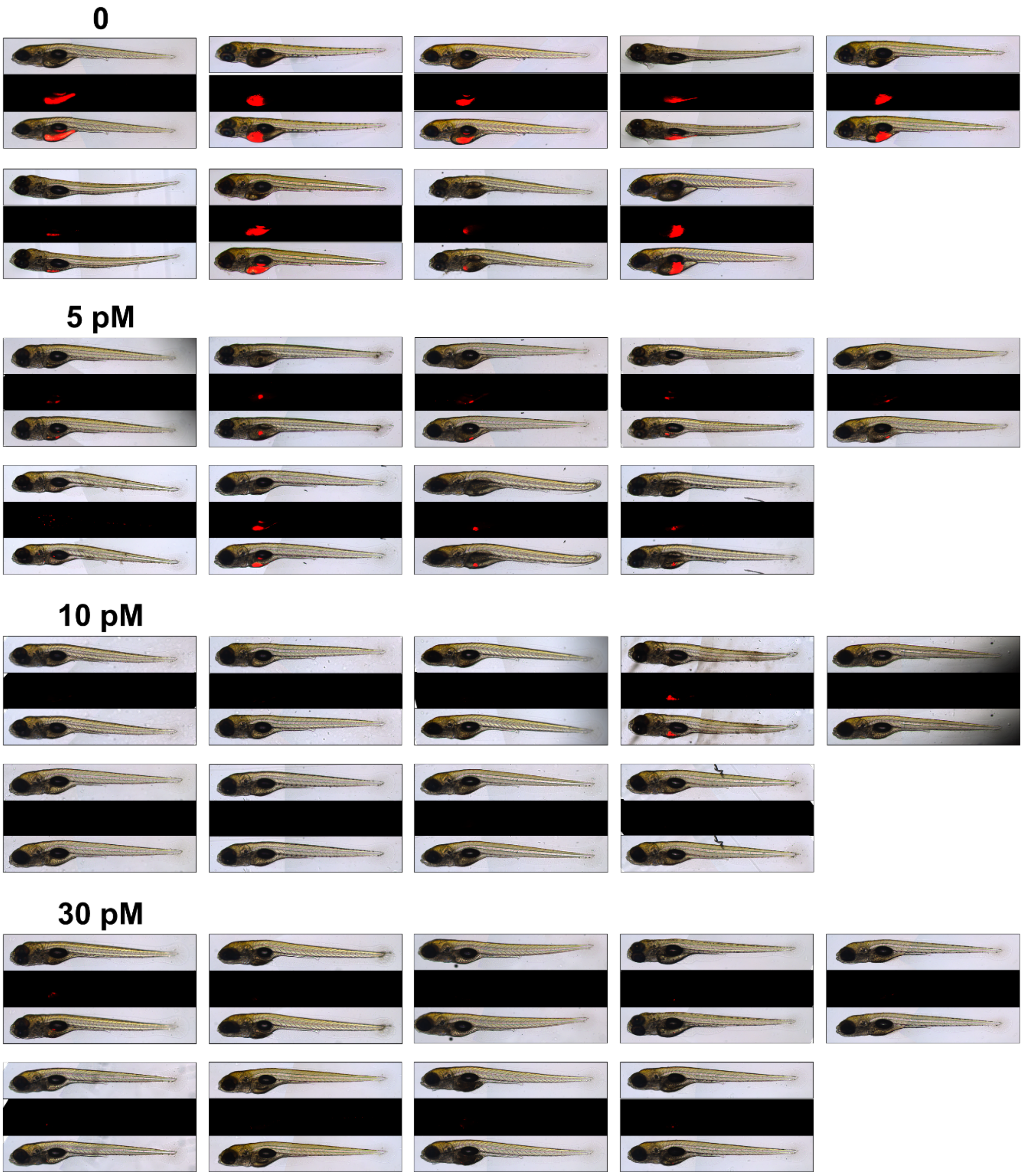

D. THP1

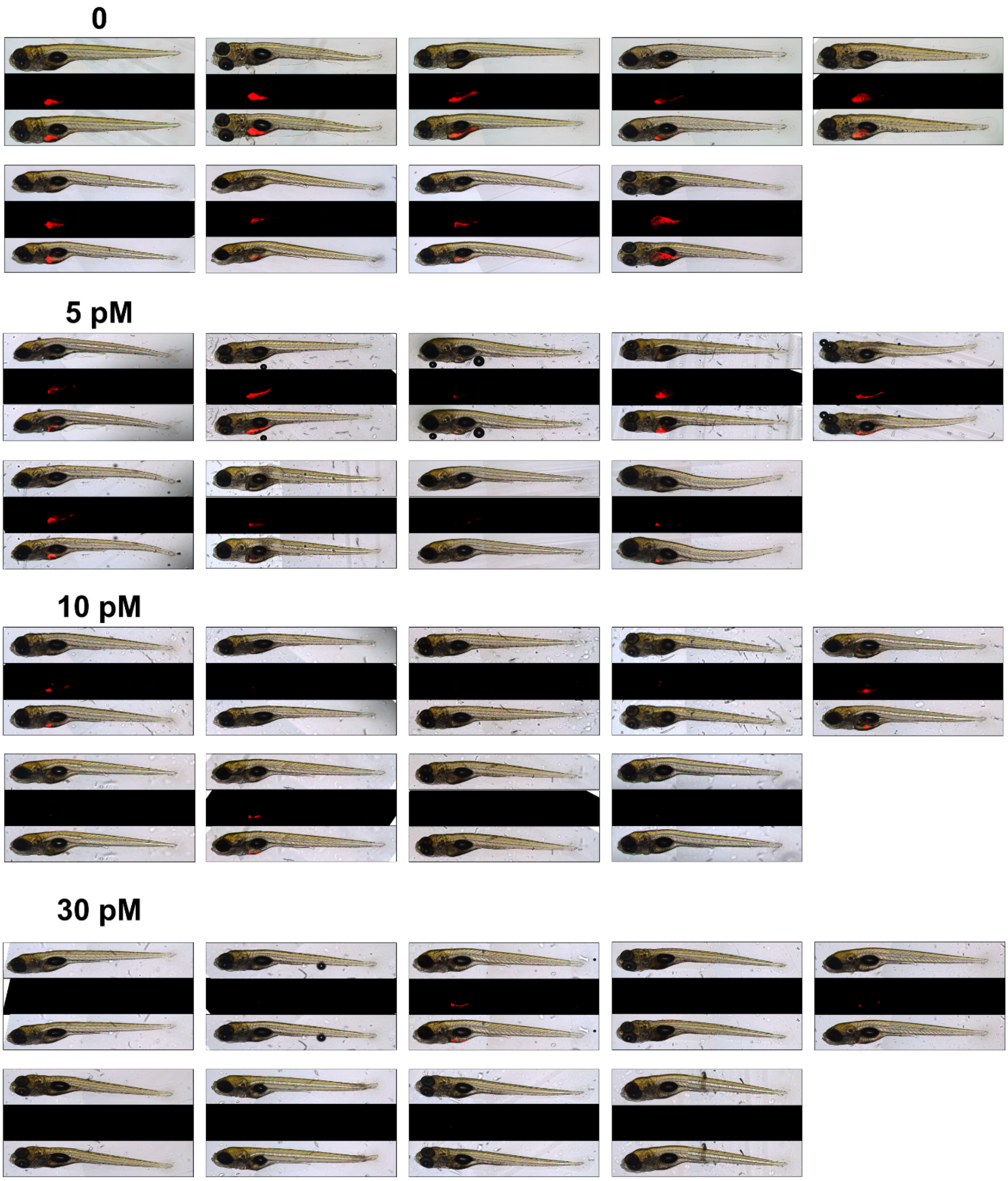

E. K562

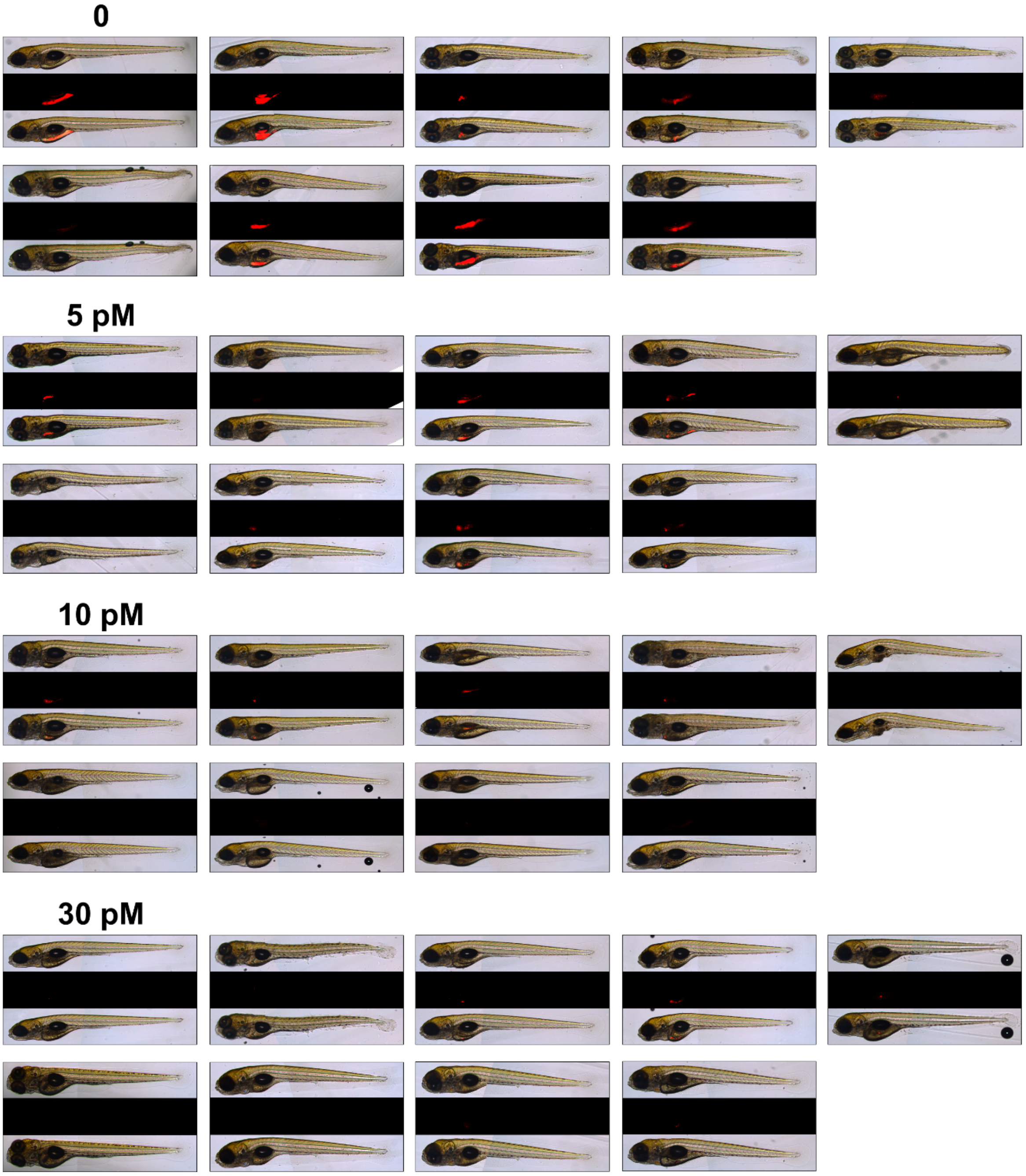

F. K562IR

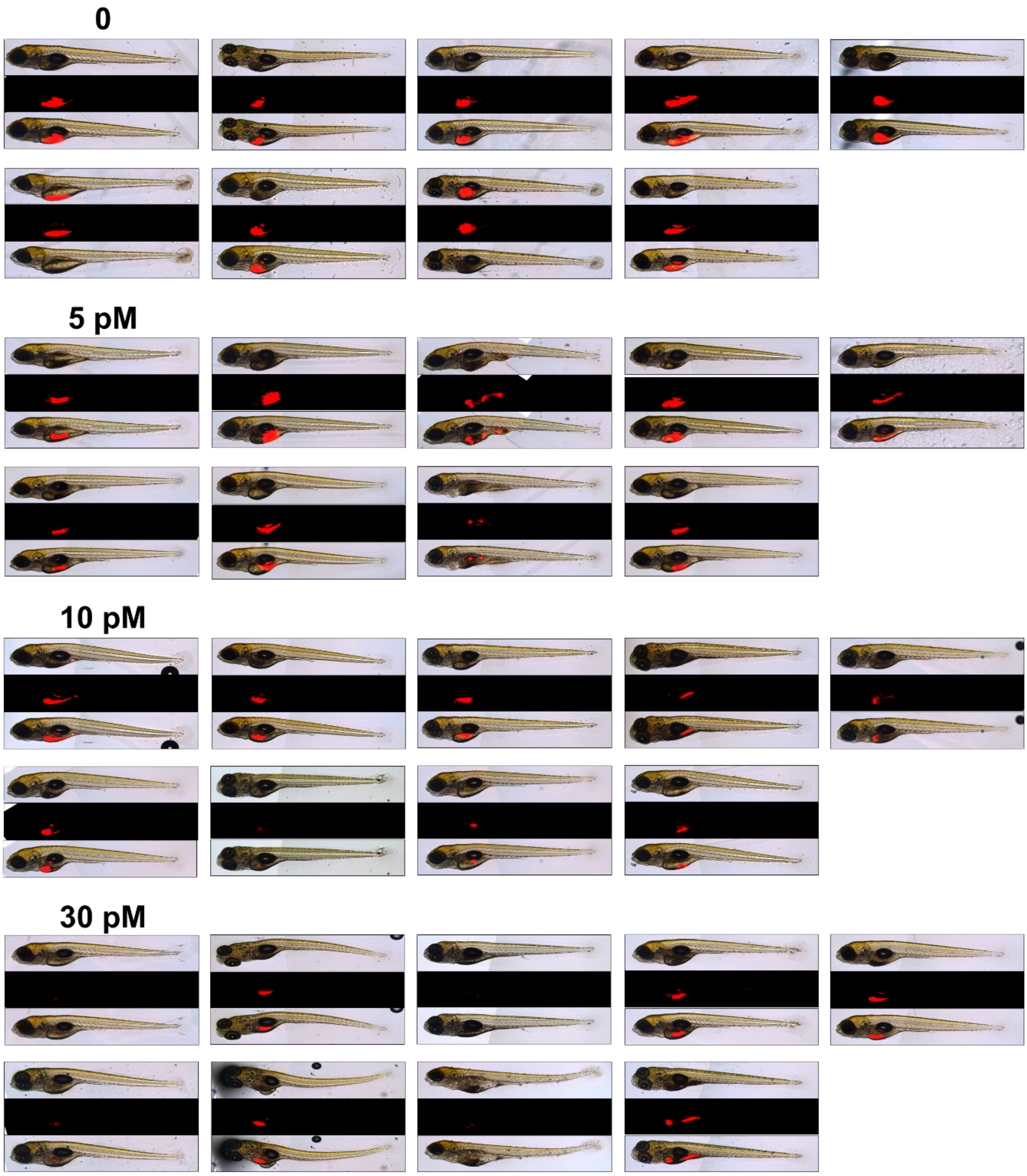

Supplement: Supplementary file 1 [file marinedrugs-21-00233-s001.zip › marinedrugs-2300114-supplementary.pdf]
